# Gonorrhœal Arthritis, Gout and Rheumatoid Arthritis

**Published:** 1890-12-13

**Authors:** 


### The Practitioner's Mirror and Retrospect.

[*The Editor will be glad to receive offers of co-operation and contributions from members of the profession. There is no doubt that much valuable material full of interest and instruction is at present lost to science. This is largely so in the case of* (1) *the younger and less-known members of the hospital staff* (2) *those connected with cottage hospitals, and* (3) *the great body of medical practitioners not attached to any institution. The co-operation of all is cordially invited to enable the Editor to make* The Hospital *of the utmost possible use and value to the profession. Specialists willing to review books on their special subjects are invited to communicate this fact at once. All letters should be addressed to* The Editor, The Lodge, Porchester Square, London, W.]

### THE LONDON POST-GRADUATE COURSE AT THE PADDINGTON INFIRMARY.

### GONORRHŒAL ARTHRITIS, GOUT AND RHEUMATOID ARTHRITIS.

Dr. Cheadle, after his lecture, which we reported last week, showed in the wards of the infirmary a woman suffering from acute rheumatic endo- and pericarditis with a presystolic murmur. He also showed three children, which he had brought from Great Ormond Street, illustrating rheumatic subcutaneous nodules; they all had cardiac mischief. Dr. Cheadle, having in his last lecture spoken of true rheumatism, the first of the four classes of rheumatic affections, now proceeded to consider *seriatim* the other three classes, namely: gonorrhœal arthritis, gout and rheumatoid arthritis. He commenced with *gonorrhœal arthritis*, by some spoken of as gonorrhœal rheumatism. Some of the authorities maintain that this is a true rheumatism modified by gonorrhœa, that is a rheumatism stirred up by gonorrhœal poison. Cases do occur in which this form is not distinguishable from ordinary attacks of acute rheumatism, and such cases are much relieved by salicylates of soda. There was a case at the present time in the wards of the infirmary of a man who had had some eleven attacks of rheumatism, all of which he referred to a gleet. The first attack was unusually severe; there was much sweating, and many joints were affected. Now he had a double aortic murmur, and probably an adherent pericardium. These cases may be coincidents, or may be due to the poison set up by the gonorrhœa. He was disposed to believe rather that an attack of general rheumatism might stir up a mucous membrane which had been rendered sensitive by previous inflammation, *i.e.*, a rheumatic blenorrhœa. The specific micro-organism, the gonococcus, has been found in the joints in some cases of gonorrhœal arthritis. This form differs from ordinary rheumatism in the following points: In the first place, the constitutional symptoms are not so severe; also the pyrexia is slight, and hyper-pyrexia is unknown. The disease also differs from acute rheumatism in its less tractability to treatment: salicylate of soda or many other drugs may be given without much effect. So also the synovial effusion is greater, the swelling of the tissues less, cardiac affections are rare, and, if present, are of a septicæmic nature. The liability for the disease to attack the lower extremities, the knees and toes, rather than the upper. Gonorrhœal arthritis more resembles septicæmia in its nature; but the serous membranes, the pericardium, and the pleura escape.

He next considered *Gout*, which is the third member of this class. The influence of the disease is not only more widely distributed than the previous one, but also almost every disturbance of any organ has been by some authorities ascribed to gout. There is no doubt the influence of the poison is very widespread, and that it affects all the organs, not only the cartilages, but also the glands, liver, kidneys, lungs, &c. It differs essentially from true rheumatism; in fact, the only point of resemblance is the occurrence of arthritis. Gout also differs from rheumatism pathologically. It is almost unknown in childhood, whereas rheumatism first appears and is most disastrous at that period. Cases of reported gout in childhood are probably rheumatic, or some are possibly of a scorbutic nature. Yet the children of gouty parents are often liable to another gouty affection—namely, hepatic disturbances and to the passing of uric acid in the urine. Gout in its onset differs from rheumatism; in gout the onset is very sudden, and there is a tendency to the affection of the smaller joints. There is intense pain, rapid swelling and blueness of the joint, followed by desquamation, also the presence in the joint of deposits of urate of soda. The pyrexia is much more moderate, it is rarely above 102, the highest recorded case is 104, also the amount of fever is proportional to the local signs; whereas in rheumatism with slight local signs there may be very high fever. Again, the improbability of heart affection in acute cases, a chronic change in the heart may occur, but acute peri- and endocarditis is unknown in gout. There is also the influence on the vessels producing a gouty arthritis and phlebitis. The question arises, How much depends on the gouty poison itself, and how much on the lithæmic condition of the blood? In rheumatism, an acute inflammation of the serous membrane occurs; whereas in gout chronic degenerations of various kinds come on. Gout is of great interest in connection with the deposit of urate of soda in the joints. In connection with this subject there are many theories; one is that, in consequence of the gouty influence, there is a precipitation of the crystals of urate of soda in the joints, the presence of which sets up the inflammation. This is the view maintained by Sir Alfred Garrod, and Sir William Roberts has shown that urate of soda crystallises more readily in synovial fluid than in blood serum. Another theory is that the lithæmic blood in circulation sets up the local influences, and that there is a rapid precipitation of an irritant salt. In favour of this view two facts might be mentioned: the one that gout manifests itself with a very sudden onset, and the other that it tends to come on under circumstances favourable to the precipitation of crystals. It first attacks the lower regions of the body—namely, the foot and toe—and non-vascular parts—namely, cartilage and tendon. It also comes on at night and in cold weather. All of these are factors suggestive of the presence of a retarded circulation. So also its liability to occur in an injured joint. Sir William Roberts suggests the possibility that not only the affection of the joints, but also the neuroses and the thromboses may be due to a minute deposit of crystals in the substance of the organs or in the blood itself.

It has been proved that uric acid is precipitated from the blood when it reaches the solution of 1 in 5,714; and that uric acid begins to precipitate from blood serum as soon as it reaches 1 in 5,000. From this it follows that there must be many people frequently on the verge of getting an attack of acute gout. Three factors have been named with regard to the cause of the disease, namely: excessive production, defective secretion, and hereditary predisposition. The excessive production of uric acid used to be believed to be due to a deficient oxidation of the nitrogenous matter taken in the food; that is, it was sub-oxidized into uric acid instead of being oxidized into urea. More recently the disease has been referred to an excessive ingestion of nitrogenous material. The chief influence of a nitrogenous diet is to directly lessen the alkalinity of the blood. Undoubtedly hereditary predisposition plays an important *rôle*, so also the presence of lead in the system. In hereditary cases the first attack may come on quite late in life. He had seen such a case in a patient 75 years of age, and a case has been reported as having appeared at the advanced age of 90 years. The following is an example: A patient with a strong hereditary tendency, but one who lives carefully, gets a sore throat. She is ordered port wine and beef-tea, with the result that an attack of gout follows the dieting. With regard to the association between lead and gout, it has been ascertained that the excretion of uric acid is diminished by the exhibition of lead. Yet gout does not occur in workers in lead in all parts of the country alike, being common in the South of England, and much rarer in the North and in Scotland. In lead gout the arthritis is less acute. An important point is that a patient with lead gout is very liable to interstitial nephritis, with albuminuria.

With regard to the last of the group, namely, *Rheumatoid arthritis*, this comes on as a complement to the other forms of rheumatic affections. It is not truly a general disease, and does not affect the other organs, but only the joints. There is no pyrexia, except in rare cases. It is essentially a defective joint structure, and follows other joint lesions, especially gout. Depressing causes have an influence in its occurrence. A large number of joints are usually affected. It is, as a rule, a disease of early middle life, but may occur in childhood. It frequently occurs in women of gouty families. In conclusion, some remarks were made on the treatment of the diseases, especially with regard to the action of certain drugs on one form and not on another.

After the lecture, Dr. Cheadle went into the wards of the infirmary and demonstrated the following cases, namely: a man who had had many attacks of rheumatism associated with chronic gleet, a man with general acute gout, another with chronic gout, and a case of lead gout, also a woman with general rheumatoid arthritis.

